# P136: Multiple strategic approaches to enhance and sustain the hand hygiene compliance

**DOI:** 10.1186/2047-2994-2-S1-P136

**Published:** 2013-06-20

**Authors:** TK Leung

**Affiliations:** 1 Infection Control, Prince of Wales Hospital, Hong Kong, China

## Introduction

Hand hygiene (HH) is the single most important practice to prevent hospital acquired infections. Indeed, HH promotion is one of the important tasks for infection control team (ICT). However there is no single mean to enhance and sustain the HH compliance for the staff.

## Objectives

To share and prove multiple strategic approaches can enhance and sustain hand hygiene compliance.

## Results

Prince of Wales Hospital (a teaching hospital of the Chinese University of Hong Kong) has adopted WHO HH audit protocol since 2007. Initially the HH compliance rate was 37%. In the past few years, HH promotion relied mainly on poster and through the reminder in different hospital meeting.

Since 2011, ICT launched a series of promotion program with more structural and strategic approaches. Apart from posters, banners, foam board stands and screen savers in clinical computer; ICT increased the frequency of HH audit from yearly to monthly. The reports were sent to each departments with breakdown of compliance rate by ward. To address the problem of poor compliance of doctor, name based audit with monthly feedback to their department heads. This name based audit also applied to staff group with low compliance rate in specific departments until their compliance rate improved.

In addition, ICT provide positive reinforcement; badges or small gifts were given to staff who performs well in hand hygiene compliance. The overall HH compliance was 86% in 2012.

## Conclusion

In order to enhance staff’s awareness and change of their behavior in HH practice, multiple strategic approaches were necessary and proved to success.

## Disclosure of interest

None declared

